# Origin-Independent Dynamic Polarizability Density from Coupled Cluster Response Theory

**DOI:** 10.1021/acs.jctc.3c00753

**Published:** 2023-10-05

**Authors:** F. F. Summa, J. H. Andersen, P. Lazzeretti, S. P. A. Sauer, G. Monaco, S. Coriani, R. Zanasi

**Affiliations:** † DTU Chemistry, Technical University of Denmark, Kemitorvet Bldg. 207, DK-2800 Kongens Lyngby, Denmark; ‡ Dipartimento di Chimica e Biologia “A. Zambelli”, Università degli Studi di Salerno, via Giovanni Paolo II 132, 84084 Fisciano, SA, Italy; ∥ Department of Chemistry, University of Copenhagen, Universitetsparken 5, DK-2100 Copenhagen Ø, Denmark

## Abstract

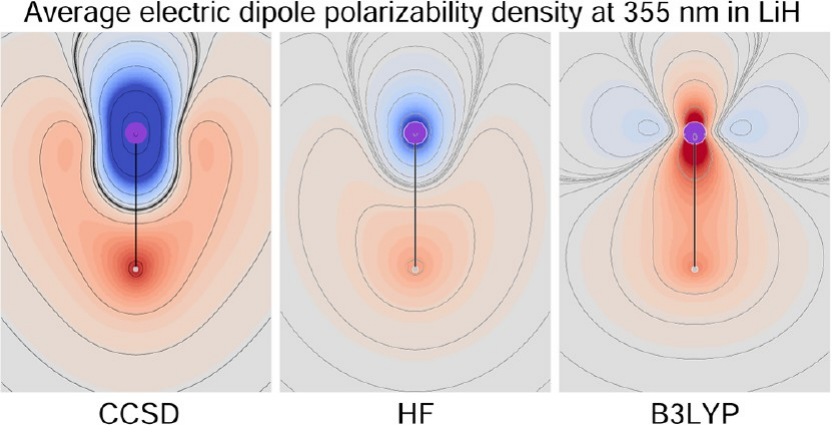

The calculation of the origin-independent density of the dynamic electric dipole polarizability, previously presented for uncorrelated and density functional theory (DFT)-based methods, has been developed and implemented at the coupled cluster singles and doubles (CCSD) level of theory. A pointwise analysis of polarizability densities calculated for a number of molecules at Hartree–Fock (HF) and CCSD clearly shows that the electron correlation effect is much larger than one would argue considering the integrated dipole electric polarizability alone. Large error compensations occur during the integration process, which hide fairly large deviations mainly located in the internuclear regions. The same is observed when calculated CCSD and B3LYP polarizability densities are compared, with the remarkable feature that positive/negative deviations between CCSD and HF reverse sign, becoming negative/positive when comparing CCSD to B3LYP.

## 1 Introduction

The concept of polarizability density α_*αβ*_(***r***), corresponding to the macroscopic polarizability tensor α_*αβ*_, was introduced in 1964 by Maaskant and Oosterhoff,^1^ as a generalization of tensors introduced by Born in his theory of optical rotatory power.^2^ These authors acknowledge similar investigations carried out by Terwiel and Mazur^3^ and reported in Terwiel’s thesis.^4,5^

The related idea of nonlocal polarizability density,^1^ α_*αβ*_(***r***, ***r***′, ω), characterized by the Born symmetry,^2^ α_*αβ*_(***r***,***r***′, ω) = α_*βα*_(***r***′, ***r***, ω), accounts for the ω-frequency component of the polarization induced at point ***r*** in a molecule by an external electric field ***E***(***r***′,*t*) acting at the point ***r***′. The nonlocal polarizability density was employed to assess the role of local fields and interparticle pair correlations in light scattering by dense fluids.^6^ Several studies by Hunt and co-workers demonstrated its utility for rationalizing van der Waals^7,8^ and short-range interactions,^9^ vibrational Raman bands,^10,11^ vibrational circular dichroism, and electric field shielding tensors,^12^ as well as force balance and force relay in molecular interactions.^13^

One year after the publication of Maaskant and Oosterhoff’s work,^1^ a paper by Theimer and Paul also reported a discussion of the static polarizability density **α**(***r***) in atoms, where the average polarizability was evaluated by integration, i.e., **α** = ∫**α**(***r***)*d****r***,^14^ while the connection between macroscopic polarizability tensor and static nonlocal polarizability density can be expressed by α_*αβ*_ = ∫α_*αβ*_(***r***, ***r***′)*d****r****d****r***′.^9^

The notion of a polarizability density tensor was afterward endorsed by other authors^15−18^ and critically examined by Sipe and Van Kranendonk^19^ and by Jameson and Buckingham,^20^ who pointed out its limitations. The fundamental objection raised by Jameson and Buckingham^20^ is that the electric polarizability density function employed in actual computations is explicitly dependent on the choice of origin. Instead, as emphasized by the authors, a fundamental requisite for the physical reliability of property densities is invariance under arbitrary translations of the reference frame. Of course, such a problem does not occur for properties depending on the difference of coordinates, e.g., the magnetic shielding density at a nucleus.^21^ Nonetheless, it clearly appears in relationships that define computational prescriptions for the polarizability density; see, for instance, eq 17 in ref (20).

Subsequently, polarizability densities of simple one-electron atoms were studied by Orttung^22^ and Orttung and Vosooghi,^23^ and the anisotropic polarizability density in the H_2_^+^ molecule was discussed by Drum and Orttung.^24^ Further investigations have been presented, taking into account also hyperpolarizability densities, via computational methods proposed by Nakano et al.,^25−27^ by Yamada et al.,^28^ and more recently by Alparone^29^ and by Otero et al.^30^ As an example, the work of Otero et al.^30^ allows for a partition of the origin-dependent polarizability density into two terms: an “intrinsic” term, containing the density matrix derivative with respect to the corresponding electric field and a relative origin with respect to each atom of the molecule, and a second term, which is size- and origin-dependent. In our opinion, none of these references report fully satisfactory proposals for computing polarizability densities that are really invariant of origin. Thus, a much-investigated problem, yet unsolved to a satisfactory extent in the theory of electric polarization, is still that of determining the main contributions to the electric dipole **μ**(ω,*t*) induced by optical ***E***(ω,*t*) in relation to different regions of a molecule. In this context, the idea of the polarizability density as a tensor function of position ***r*** comes into play, since it allows detection of the electronic polarization at each point of the electron distribution. If maps of such a tensor function were available, then, by means of suitably chosen integration domains, it would be possible, for instance, to evaluate atomic or group contributions and verify their transferability from one molecule to another.

A novel approach to the origin-independent electric dipole polarizability density, both static and dynamic, has recently been proposed based on the current density induced by the time derivative of the electric field ***E***(ω,*t*) carried by a monochromatic wave of frequency ω.^31−33^ Such a current density is a member of a large family of linear properties connected to a set of density tensor functions, each representing a specific molecular response independent of the external perturbation. In the present case, we deal with a current density tensor (CDT), invariant with respect to the translation of the origin, which gives the electric polarizability tensor in the mixed dipole length-dipole velocity formalism after integration.

The possibility of analyzing the molecular space in a punctiform way, offered by the availability of reliable density functions, opens a new perspective for the study of the electron correlation effect. Further, it allows for a meaningful comparison among different levels of approximation, in particular, with respect to the popular density functional theory (DFT)-based methods. Therefore, the present work has at least three main motivations that we would like to highlight: (i) implementation and calculation of origin-independent density of dynamic polarizabilities at the CCSD level of theory, which is, to the best of our knowledge, the first application of this kind; (ii) inspection of electron correlation effects on the calculation of the electric polarizability by comparison with the reference HF level (e.g., are there deeper effects hidden by integration?); and (iii) examination of the polarizability densities obtained with one of the most popular DFT functionals, namely, B3LYP,^34,35^ to determine the regions of the molecular space that most deviate from the CCSD determination (it would be very appealing to find indications on how to improve functionals). Since the electric polarizability in the mixed dipole length-dipole velocity formalism is identical to the conventional polarizability only if the off-diagonal hypervirial relation is satisfied, much attention has been given to verifying to what extent this condition is met (in practice) by the CCSD approximation, which notoriously presents difficulties in this sense.^36,37^

The article is organized as follows: Section 2 is devoted to a description of the essential elements of the polarizability density. Details about its implementation at the HF and DFT levels of theory are given in Section 3. Section 4 describes the CC linear response theory developed for the calculation of the perturbed density function. Results obtained for a few simple linear molecules are presented and discussed in Section 6 after a description of the computational details is given in Section 5.

## 2 Outline of Notation and Theoretical Methods

Within the Born–Oppenheimer (BO) approximation,^38^ for a molecule with *n* electrons and *N* clamped nuclei, charge, mass, position, and canonical and angular momentum of the *k*th electron are indicated in the configuration space by −*e*, *m*_e_, ***r***_*k*_, ***p*^**_*k*_ = −iℏ***∇***_*k*_, ***l*^**_*k*_ = ***r***_*k*_ × ***p*^**_*k*_, *k* = 1, 2, ···, *n*, using boldface letters for electronic vector operators. Analogous quantities for nuclei are *eZ*_*I*_, *M*_*I*_, ***R***_*I*_, etc., for *I* = 1, 2, ···, *N*. Capital letters denote total (*n*-electron) vector operators, e.g.,
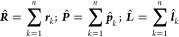
Thus, a Cartesian component of the electric dipole operator in dipole length formalism becomes μ̂_α_ = −*eR̂*_α_ (throughout, the Greek subscripts α and β will be used to indicate Cartesian components of a given operator, vector, or tensor). Expressions for the polarization charge density and current density induced in the electrons of a molecule by a monochromatic plane wave are obtained by time-dependent quantum mechanical perturbation theory,^39^ assuming that the eigenvalue problem for the time-independent BO electronic Hamiltonian, *Ĥ*^(0)^Ψ_*j*_^(0)^ = *E*_*j*_^(0)^Ψ_*j*_^(0)^, has been solved, determining a set of eigenfunctions Ψ_*j*_^(0)^ and corresponding energy eigenvalues *E*_*j*_^(0)^.

The reference (ground) state is indicated by Ψ_*a*_^(0)^, and the natural transition frequencies are ω_*ja*_ = (*E*_*j*_^(0)^ – *E*_*a*_^(0)^)/ℏ. We introduce the general definition of the *n*-electron probability density matrix^40^ for a state function Ψ(***X***)
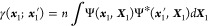
1of electronic space-spin coordinates ***x***_*k*_ = ***r***_*k*_ ⊗ η_*k*_, *k* = 1, 2, ···, *n*, where
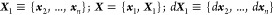
2By integrating over the spin variable η_1_, a spatial probability density matrix is obtained
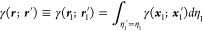
3Putting ***r*** = ***r***′, we obtain the probability density
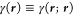
4For the reference (ground) state Ψ_*a*_^(0)^, probability and charge densities are
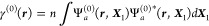
5
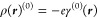
6

A beam of monochromatic light of frequency ω induces oscillating charge and current distributions in the electron cloud of a molecule. These periodic oscillations can be expressed in terms of dynamic electric polarizabilities and hyperpolarizabilities, i.e., response tensors of increasing rank, explicitly depending on ω. Within the long-wavelength approximation,^41,42^ the main contribution to light scattering is provided by the oscillating electric dipole **μ**(ω,*t*) induced by the electric field of the monochromatic wave, whose strength ***E***(ω,*t*) = ***E***_0_cos(*ωt*) is assumed to be spatially uniform all over the molecular domain. If we limit ourselves to consider a linear response, the induced dipole is expressed by the following relationship (Einstein summation convention over repeated indices is implied here and throughout)
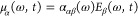
7where the second-rank polar tensor α_*αβ*_(ω), symmetric under the exchange α ↔ β, represents the frequency-dependent electric dipole polarizability.

If the intensity of the optical field ***E***(ω,*t*) of impinging radiation is weak, first-order time-dependent perturbation theory^39^ can be applied to describe the interacting system. Accordingly, the total electronic charge density is expressed via a truncated series
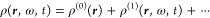
8where
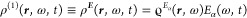
9introducing the vector function
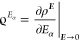
10which characterizes the charge polarization to first order. For any value of ω, including ω = 0 for a static electric field, the charge polarization vector function is cast in the following form^32^
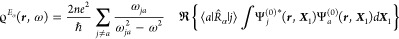
11where ⟨*a*| ≡ ⟨Ψ_*a*_^0^| and |*j*⟩ ≡ |Ψ_*j*_^0^⟩.  stands for the real/imaginary part of the quantity within curly brackets. To the first order, the electric dipole moment (eq 7) induced in the electron distribution is given by
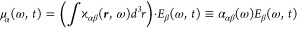
12where (***r***, ω) is a polarizability density tensor function defined by the following relationship
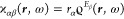
13which is not symmetric in the α and β indices, and
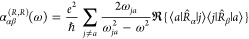
14is the frequency-dependent electric dipole polarizability in the dipole length gauge, symmetric in the α and β indices.^43^

Owing to the orthogonality of the eigenstates Ψ_*a*_^(0)^ and Ψ_*j*_^(0)^, the space integral of the polarization density vector (eq 11) over the entire molecular domain vanishes, thus fulfilling the constraint of charge conservation.^32^ Therefore, the induced dipole moment (eq 12) is independent of the origin of the *r*_α_ vector. Accordingly, no origin is specified in this equation. However, the components of the position vector ***r*** depend on the origin ***r***′ chosen for the coordinate system and change in a passive parallel translation represented by an arbitrary shift ***d***
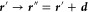
15Therefore, the polarizability density in eq 13 varies in plots obtained using different coordinate systems.^33^ For this reason, visualizations reported so far for the polarizability density tensor based on eqs 11–13 are of dubious physical meaning and computationally impractical.

A more promising approach is available within the framework of the origin-independent current density vector ***J***^***Ė***^, induced by the time derivative ***Ė***(ω,*t*) of the electric field of monochromatic radiation.^31,44^
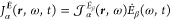
16Thus, more viable computational procedures are based on dynamic, second-rank CDT obtained by differentiation of the current density vector
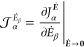
17expressed in the following form^31^
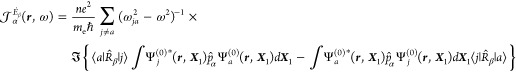
18

The translationally invariant CDT defined via eq 18 can be interpreted as a polarizability density function,^32^ alternative to—and, from the physical viewpoint, more meaningful than—the widely adopted expression in eq 13, since
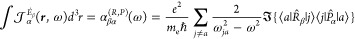
19where α_*βα*_^(*R*,*P*)^(ω) is the frequency-dependent electric dipole polarizability in mixed dipole length-dipole velocity formalism. For a complete derivation of the expressions here reported, see ref (45) and the Supporting Information attached. α_*βα*_^(*R*,*P*)^(ω) is identical to eq 14 if the off-diagonal hypervirial relationship^46,47^
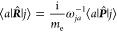
20is satisfied. Incidentally, it is worth recalling that off-diagonal α_*βα*_^(*R*,*P*)^ tensor components are symmetric in the exchange α ↔ β only if eq 20 is fulfilled, i.e., in the case of exact eigenfunctions to a model Hamiltonian and optimal variational eigenfunctions.^48^ Within the algebraic approximation,^49^ in the absence of molecular point group symmetry, the identity α_*βα*_^(*R*,*P*)^ = α_*αβ*_^(*R*,*P*)^ is satisfied only in the limit of a complete basis set. The degree to which it is fulfilled, together with α_*αβ*_^(*R*,*P*)^ = α_*αβ*_^(*R*,*R*)^, provides simple tests for basis set quality.^50^

The densities (eqs 9 and 16) are connected to one another by the continuity equation^51^
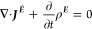
21which, allowing for eqs 11 and 18, becomes the vector equation
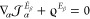
22

The great advantage offered by the definition given in eq 18 with respect to eq 13 is immediately evident in that it is invariant of the origin. It is valid for any value of ω, including ω → 0, i.e., for a static electric field. Plots of the density (eq 18) provide fundamental information on the polarization of the electron cloud.^33^

The tensor  is connected with two physical quantities, depending on whether it is multiplied by ***E***(ω,*t*) = ***E***_0_ cos(*ωt*) or ***Ė***(ω,*t*) = ω***E***_0_ cos(*ωt* + π/2), i.e.,
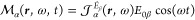
23
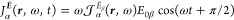
24These relations^44,52^ define the induced dipole density  and current density vector ***J***^***E*˙**^(***r***,ω,*t*). In the static case, we have ***E***(0, *t*) = ***E***_0_ and ***Ė***(0, *t*) = **0**, and visualizations of the polarizability density provide information on the dipole moment induced in the molecule. For ω ≠ 0, the oscillating charge is described by the current density vector induced by an electric field out of phase of π/2.

## 3 Implementation of the Polarizability Densities at HF and DFT Level of Theory

The theoretical formulation of the polarizability density function described above can be straightforwardly implemented within the random phase approximation (RPA) formulation of the time-dependent Hartree–Fock (TD-HF)^53−55^ and time-dependent density functional theory (TD-DFT)^56−59^ frameworks. The present section aims to discuss how the implementation of the polarizability densities, in length and in mixed length–velocity gauges, can be achieved starting from the definitions of ϰ_*αβ*_ and  given in eqs 13 and 18. Using a notation similar to the one adopted before, eqs 13 and 18 are rewritten in the following forms
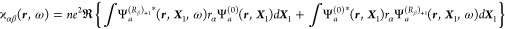
25
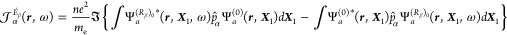
26where
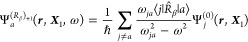
27
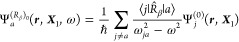
28are regarded as vectors explicitly depending on the radiation frequency ω. For an unrestricted open-shell system, represented by a one-determinant wave function
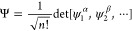
29constructed by *n* = *n*_α_ + *n*_β_ occupied molecular orbitals, the previous equations become, in atomic units, respectively
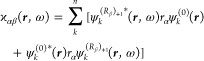
30
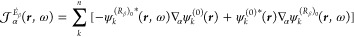
31Then, spin–orbitals ψ_*k*_ are expanded as linear combinations of basis functions χ_γ_
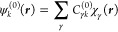
32
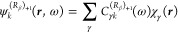
33
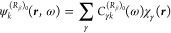
34where *C*_*γk*_^(0)^, *C*_*γk*_^(*R*_β_)_+1_^(ω), and *C*_*γk*_^(*R*_β_)_0_^(ω) are unperturbed and perturbed coefficients that are different for α and β spin–orbitals. The coefficients can be obtained using the RPA at the HF or DFT level of theory. To be complete and also to extend the results to all kinds of electronic structure calculation methods, we can introduce the equations rewritten using the density matrices approach^40^
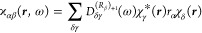
35
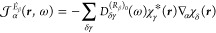
36where, for real coefficients, the symmetric density matrix *D*_*δγ*_^(*R*_β_)_+1_^(ω) is defined as
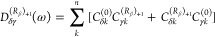
37and the antisymmetric one *D*_*δγ*_^(*R*_β_)_0_^(ω) as
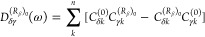
38with coefficients defined to be
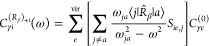
39
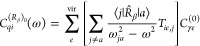
40In our implementation, the transition amplitudes ***S***_*j*_ and ***T***_*j*_, and corresponding transition energies ω_*ja*_, are obtained from a TD-DFT (or TD-HF ≡ RPA) calculation. In particular, the ***S*** and ***T*** matrices are defined as in ref (55) and are determined from the standard amplitudes ***X*** and ***Y***,^57−59^ as ***S*** = √2(***X*** + ***Y***) and ***T*** = √2(***X*** – ***Y***). The utilities contained in Gaussian v. 16^60^ (to which our code is interfaced) have been used to obtain ***X*** and ***Y*** in machine precision. The full procedure for the calculation of the frequency-dependent electric dipole polarizability density has been implemented within the freely available SYSMOIC(61) program package.

## 4 Implementation of the Polarizability Densities within Coupled Cluster Linear Response Theory

From the CC asymmetric linear response function,^62^ the dynamic polarizability (in MO basis) in the length gauge can be expressed (in atomic units) as
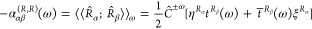
41The *Ĉ*^±ω^ operator symmetrizes the function by a change of the sign of the frequencies and complex conjugation. For the definition of the quantities entering the above and following equations, see, e.g., ref (62).

We can rewrite the response function of eq 41 in terms of perturbed density matrices and with explicit summation over MO indices *p*,*q*
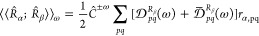
42where the perturbed CC densities are the derivatives of the CC one-electron density matrix with respect to the perturbed amplitudes and multipliers
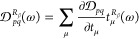
43
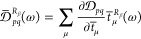
44

The densities can be transformed to the AO basis (symbols *D*_*δγ*_^*R*_β_^ and *D̅*_*δγ*_^*R*_β_^) via the MO coefficients from the linear combination *X*_*pq*_ = ∑_*δγ*_*C*_*pδ*_^T^*X*_*δγ*_*C*_*γq*_. Further introducing the *r*_α_ integrals in the AO basis from previous sections, we obtain the response function in the form
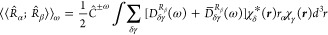
45Defining (minus) the symmetrized total density matrix as
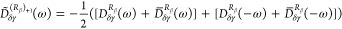
46we identify in eq 45 the CC polarizability density in the length gauge
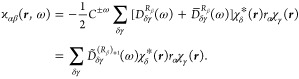
47

The mixed length–velocity CC polarizability density is obtained in an equivalent manner by making the exchange *R̂*_β_ → *i*ω^–1^*P̂*_β_ in eq 41, ultimately resulting in the expression
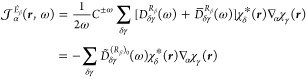
48with the antisymmetrized total density matrix
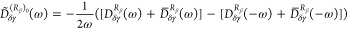
49In eq 48, the density is antisymmetrized, since the response function is imaginary.

The perturbed AO density matrices, eqs 46 and 49, have been implemented at the CCSD level of theory in our in-house Python-based prototyping CC response code pyCCRSP.^63^ Following the calculation of perturbed density matrix elements in the AO basis with pyCCRSP, their contraction with the atomic orbitals to obtain the polarizability densities according to eqs 47 and 48,47 was subsequently carried out in SYSMOIC.^64^

## 5 Computational Details

The origin-independent dynamic electric-dipole polarizability density was calculated according to the theory exposed in the previous sections at the HF, DFT, and CCSD levels of theory for a few diatomic molecules. They are H_2_, LiH, HF, LiF, N_2_, and CO, chosen for their simplicity and high symmetry. Indeed, thanks to their linear structure, only two components of the polarizability density tensor need to be examined, which makes the analysis of the results easier at this stage of development. Nonetheless, they allow us to consider different chemical bondings from pure covalent to ionic and to compare CCSD results with those obtained by adopting lower level, but much more widespread, computational methods such as B3LYP and HF.

The basis set of Gaussian functions adopted here is aug-pcSseg-2,^65^ known to provide a good compromise between the accuracy of the results and the calculation efforts. In particular, for the fulfillment of the off-diagonal hypervirial relation, which requires accurate calculations of transition dipole moments and excitation energies,^66−68^ it is important to include both diffuse and tight functions in the basis set. Since the aug-pcSseg-2 basis set was devised for shielding calculations, it contains both types and is therefore well suited for the project.

Molecular geometries have been optimized at the CCSD/aug-pcSseg-2 level of theory using the Gaussian16 program package.^60^ For all molecules, the chemical bond was set along the *z* Cartesian axis. Thus, the *zz* tensor component coincides with the parallel component, and the degenerate *xx* and *yy* tensor components correspond to the perpendicular component.

The optical wavelengths adopted in this work, commonly used also in gas-phase experiments,^69,70^ are λ = 633, 589.3, and 355 nm (corresponding to frequencies ω in au of 0.0720, 0.07732, and 0.128, respectively).

The polarizability density maps shown in the following sections were calculated for grids of points over molecular planes of the size quoted along their edges. A grid step of 0.025 au is utilized for all maps.

## 6 Results and Discussion

Fundamental for this work is the fulfillment, in a practical sense at least, of the off-diagonal hypervirial relationships, eq 20. Therefore, we start this section by examining this aspect for all three methods here employed.

### 6.1 Fulfillment of the Off-Diagonal Hypervirial Relation

As well known, the TD-HF method exactly fulfills the extra-diagonal hypervirial theorem in the complete basis set limit.^49,71,72^ Therefore, we first examined the polarizability tensor components calculated using this method, reported in Table 1 for the three selected radiation wavelengths. Polarizability components have been obtained by integrating the densities, eqs 35 and 36, corresponding to mixed dipole length-dipole velocity (*R*, *P*) and dipole-length (*R*, *R*) formalisms, respectively. In this way, it is possible to test the performance of the adopted basis set and establish a reference point for the other two methods employed here.

**Table 1:** HF/aug-pcSseg-2 Electric Dipole Polarizability (au) in Mixed Dipole Length-Dipole Velocity (*R*, *P*) and Dipole-Length (*R*, *R*) Formalisms^a^

		α_⊥_^(*R*,*P*)^			α_∥_^(*R*,*P*)^			α_⊥_^(*R*,*R*)^			α_∥_^(*R*,*R*)^		
mol	FTE	633	589.3	355	633	589.3	355	633	589.3	355	633	589.3	355
H_2_	98	4.4	4.4	4.6	6.6	6.6	6.9	4.4	4.5	4.6	6.6	6.6	6.9
LiH	307	28.7	29.3	42.2	25.7	26.5	52.6	28.8	29.4	42.4	25.7	26.4	52.5
HF	106	4.5	4.5	4.6	5.8	5.8	5.9	4.5	4.5	4.6	5.8	5.8	5.9
LiF	152	7.7	7.8	8.1	7.6	7.6	7.9	7.7	7.7	8.1	7.6	7.6	7.9
N_2_	155	9.8	9.8	10.1	15.1	15.2	15.6	9.8	9.9	10.1	15.2	15.2	15.7
CO	141	11.4	11.5	12.0	14.6	14.6	15.1	11.4	11.5	12.0	14.6	14.7	15.1

a First transition energy (FTE) is in nm. The conversion factor to SI units is *e*^2^*a*_0_^2^/*E*_h_ = 1.648777273 × 10^–41^ F m^2^.

Already at first glance, the HF method shows a remarkable concordance of the results obtained in the two formalisms, which also extends to heavier nuclei in the nitrogen and carbon monoxide molecules. On quantitative grounds, we see that the average relative absolute deviation amounts to only 0.3% for HF, which relieves any problems concerning the adequacy of the basis set employed. The maximum relative absolute deviation is about 1.5% for the perpendicular component of H_2_.

For TD-DFT, the off-diagonal hypervirial relationship is fulfilled too.^73−75^ The B3LYP polarizability components are listed in Table 2. Also, in this case, a remarkable agreement of the results for the two formalisms can be observed. On average, the maximum relative absolute deviation amounts to 0.3%, while the maximum relative absolute deviation is only 1%. Therefore, the B3LYP/aug-pcSseg-2 fulfills the off-diagonal hypervirial theorem to an extent comparable to that of the HF method.

**Table 2:** B3LYP/aug-pcSseg-2 Electric Dipole Polarizability (au) in Mixed Dipole Length-Dipole Velocity (*R*, *P*) and Dipole-Length (*R*, *R*) Formalisms

		α_⊥_^(*R*,*P*)^			α_∥_^(*R*,*P*)^			α_⊥_^(*R*,*R*)^			α_∥_^(*R*,*R*)^		
mol	FTE	633	589.3	355	633	589.3	355	633	589.3	355	633	589.3	355
H_2_	109	4.8	4.8	5.0	6.9	7.0	7.3	4.8	4.8	5.0	7.0	7.0	7.3
LiH	387	36.9	38.1	75.2	39.2	41.9	–65.2	37.0	38.2	75.5	39.1	41.8	–64.9
HF	131	5.5	5.5	5.7	6.6	6.6	6.8	5.5	5.6	5.7	6.6	6.6	6.8
LiF	234	11.1	11.2	12.7	10.9	11.0	11.8	11.1	11.2	12.7	11.0	11.0	11.9
N_2_	133	10.5	10.5	10.8	15.4	15.4	15.9	10.5	10.5	10.8	15.4	15.4	15.9
CO	148	12.4	12.4	13.1	15.7	15.7	16.3	12.4	12.4	13.1	15.7	15.8	16.4

The polarizability components calculated at the CCSD level using eqs 47 and 48, for the dipole-length (*R*, *R*) and mixed dipole length-dipole velocity (*R*, *P*) formalisms, respectively, are collected in Table 3. As can be seen, the comparison of the results obtained for the two formalisms is still rather encouraging. The largest deviations are observed for the parallel component of N_2_, followed by a smaller deviation for both components of CO. However, in comparison with those of HF and B3LYP, the agreement between the two formalisms is less satisfactory.

**Table 3:** CCSD/aug-pcSseg-2 Electric Dipole Polarizability (au) in Mixed Dipole Length-Dipole Velocity (*R*, *P*) and Dipole-Length (*R*,*R*) Formalisms

		α_⊥_^(*R*,*P*)^			α_∥_^(*R*,*P*)^			α_⊥_^(*R*,*R*)^			α_∥_^(*R*,*R*)^		
mol	FTE	633	589.3	355	633	589.3	355	633	589.3	355	633	589.3	355
H_2_	97	4.3	4.3	4.4	6.5	6.5	6.8	4.4	4.4	4.5	6.5	6.6	6.8
LiH	346	34.9	35.9	61.1	33.2	34.7	303.7	35.4	36.4	61.9	33.3	34.8	303.6
HF	119	5.2	5.3	5.4	6.4	6.4	6.5	5.3	5.3	5.4	6.4	6.4	6.6
LiF	189	10.2	10.3	11.1	9.9	9.9	10.5	10.1	10.2	11.1	9.9	9.9	10.5
N_2_	131	9.9	9.9	10.2	13.8	13.8	14.2	10.1	10.2	10.4	14.7	14.8	15.2
CO	143	11.6	11.7	12.2	15.2	15.2	15.8	12.0	12.0	12.6	15.8	15.9	16.4

We attribute the disagreement primarily to the nonvariational nature of the CCSD method and to a lesser extent to the basis set incompleteness (the H_2_ results can be used as an indicator for the latter). However, on a quantitative ground, the average relative absolute deviation remains quite small, i.e., 1.8%, while the largest relative absolute deviation is about 6% for the parallel component of N_2_, which is still reasonably satisfactory. Based on these results, we conclude that the off-diagonal hypervirial relationship (eq 20) is met in practice to an acceptable extent also by the CCSD method for the specific molecules here considered.

It is worth noting that the electric dipole polarizabilities calculated at the CCSD level lie almost in all cases between those calculated via the HF and B3LYP methods. Indeed, the polarizability tensor components estimated at the HF level of theory, see Table 1, are in general smaller than the CCSD ones, with the exception of both components of H_2_ and the parallel component of N_2_. Conversely, the polarizability tensor component computed by using the B3LYP functional is systematically larger, as shown by the results displayed in Tables 2 and 3.

### 6.2 Origin-Independent CCSD Polarizability Density

**Figure 1:**
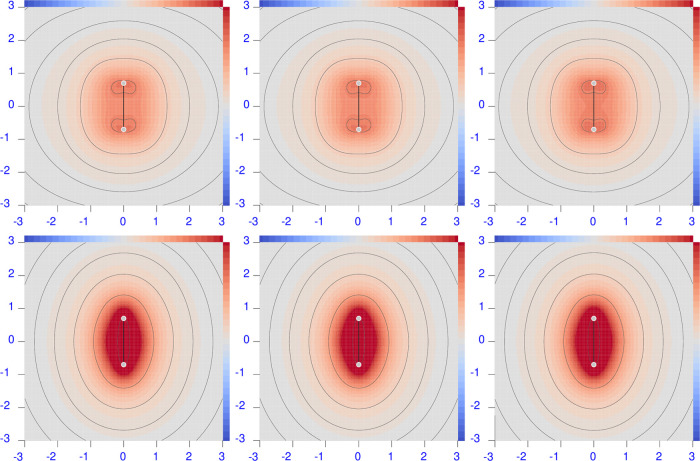
Diverging color map of the origin-independent polarizability density functions of H_2_ calculated at the CCSD/aug-pcSseg-2 level of theory (equivalent to FCI/aug-pcSseg-2). For the three radiation wavelengths λ = 633 nm (left), 589.3 nm (middle), and 355 nm (right), the top row shows the perpendicular component, and the bottom row shows the parallel one. The sidebar range is [−0.5(dark blue), 0.5(dark red)] a.u.

**Figure 2:**
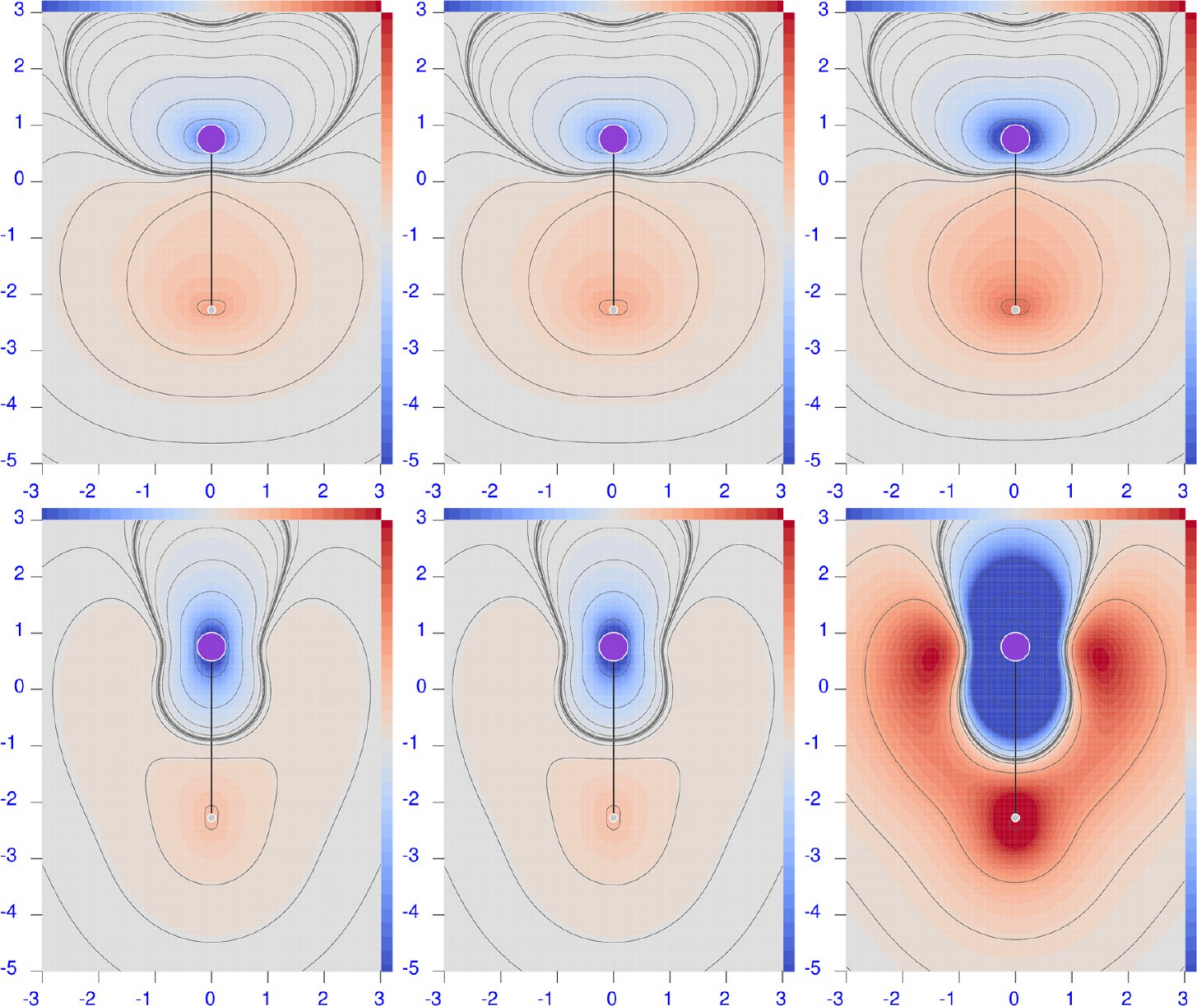
Diverging color map of the origin-independent CCSD/aug-pcSseg-2 polarizability density functions of LiH. For the three radiation wavelengths λ = 633 nm (left), 589.3 nm (middle), and 355 nm (right), the top row shows the perpendicular component, and the bottom row shows the parallel one. The sidebar range is [−4(dark blue), 4(dark red)] a.u.

**Figure 3:**
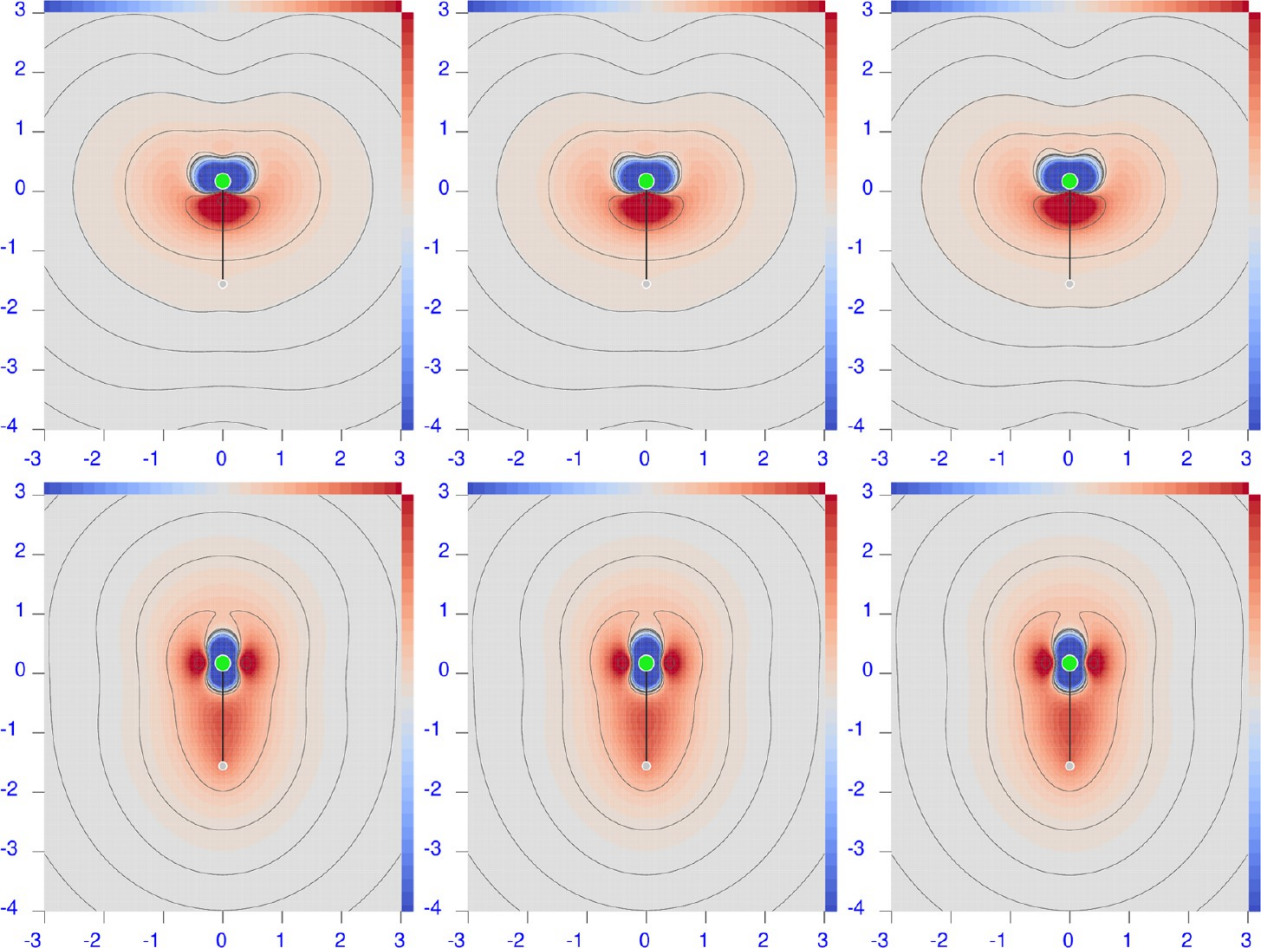
Diverging color map of the origin-independent polarizability density functions of HF, calculated at the CCSD/aug-pcSseg-2 level of theory. For the three radiation wavelengths λ = 633 nm (left), 589.3 nm (middle), and 355 nm (right), the top row shows the perpendicular component, and the bottom row shows the parallel one. The sidebar range is [−1(dark blue), 1(dark red)] a.u.

**Figure 4:**
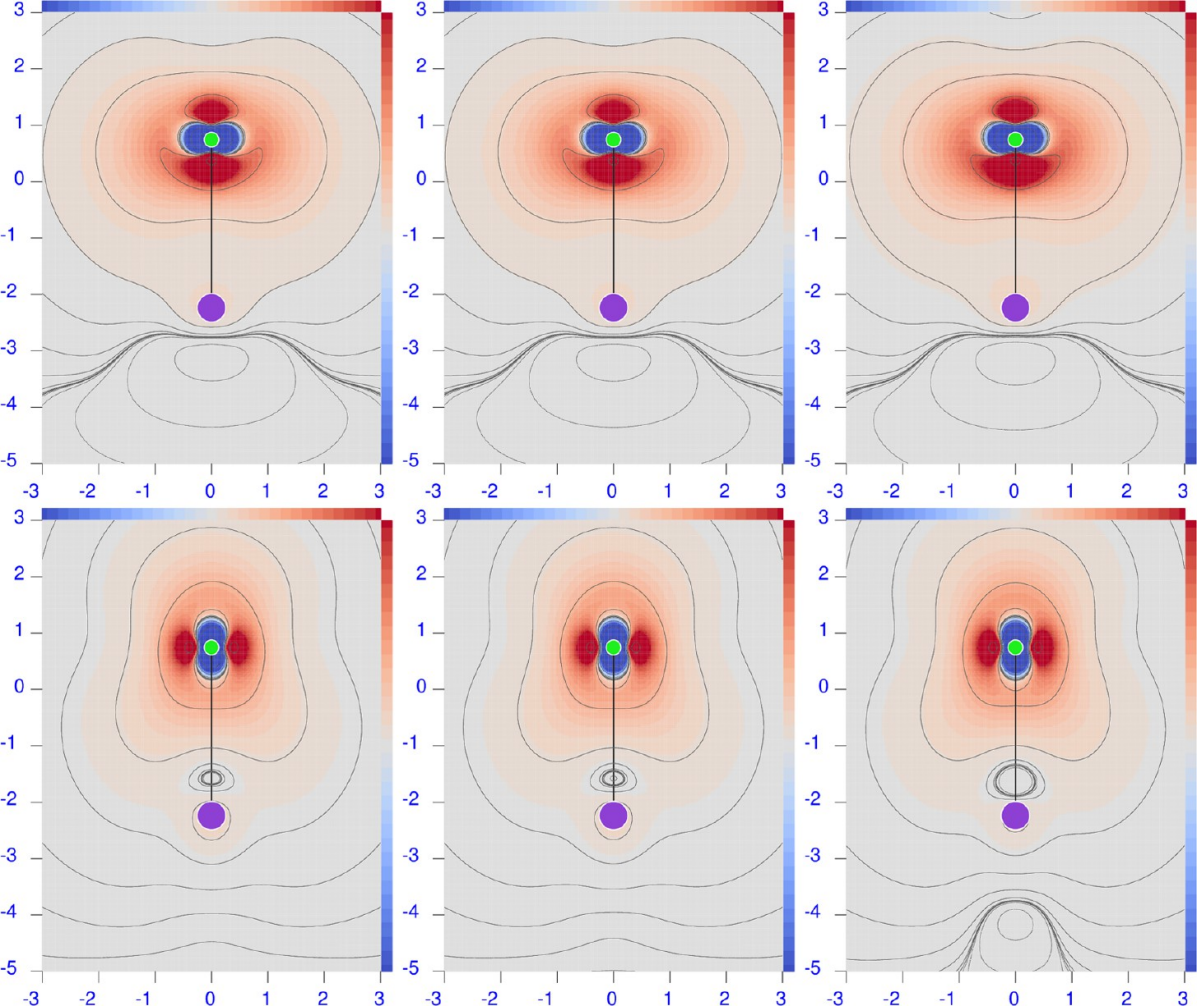
Diverging color map of the origin-independent polarizability density functions of LiF, calculated at CCSD/aug-pcSseg-2 level of theory For the three radiation wavelengths λ = 633 nm (left), 589.3 nm (middle), and 355 nm (right), the top row shows the perpendicular component, and the bottom row shows the parallel one. The sidebar range is [−1(dark blue); 1(dark red)] a.u.

**Figure 5:**
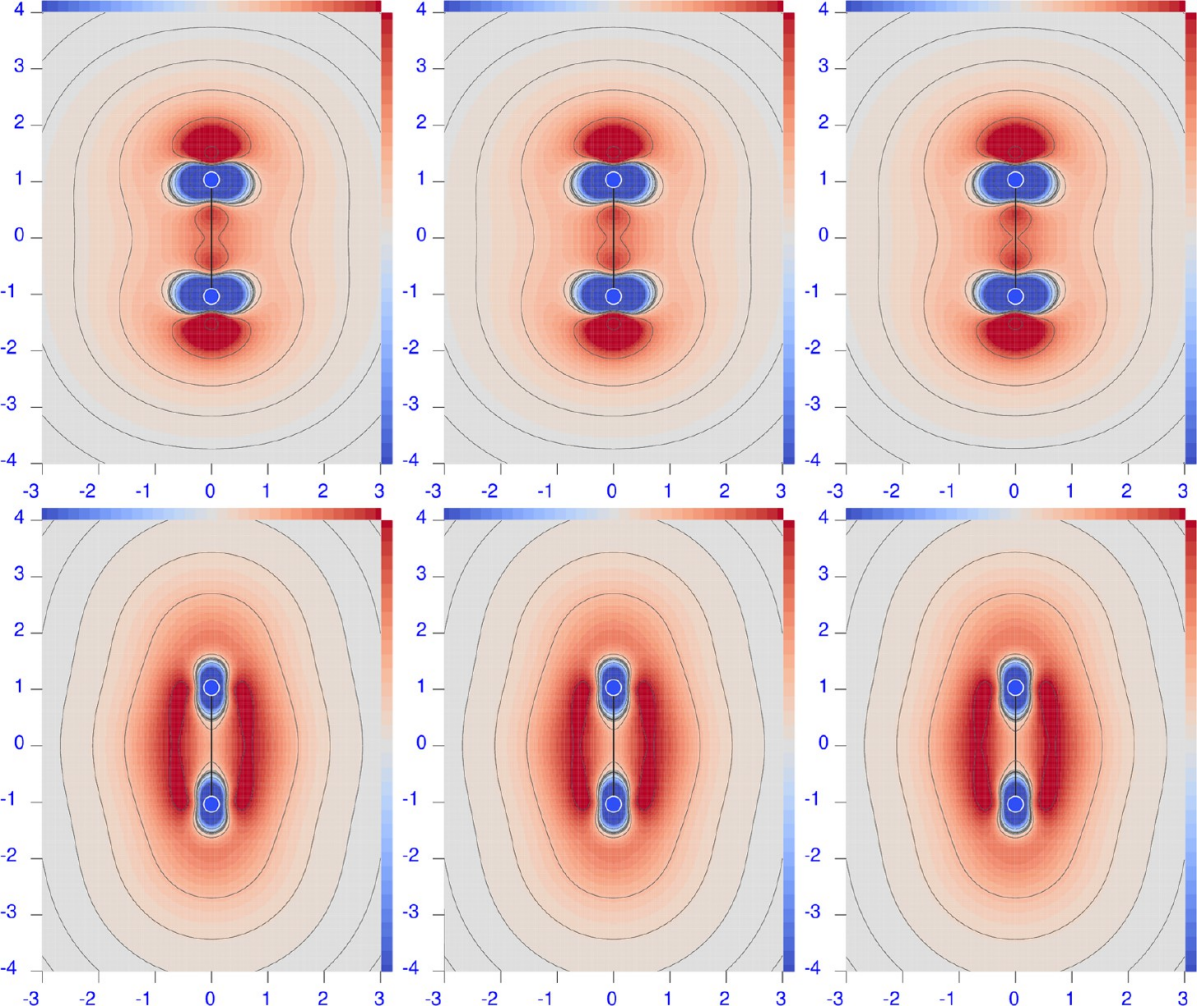
Diverging color map of the origin-independent polarizability density functions of N_2_, calculated at the CCSD/aug-pcSseg-2 level of theory. For the three radiation wavelengths λ = 633 nm (left), 589.3 nm (middle), and 355 nm (right), the top row shows the perpendicular component, and the bottom row shows the parallel one. The sidebar range is [−0.5(dark blue), 0.5(dark red)] a.u.

**Figure 6:**
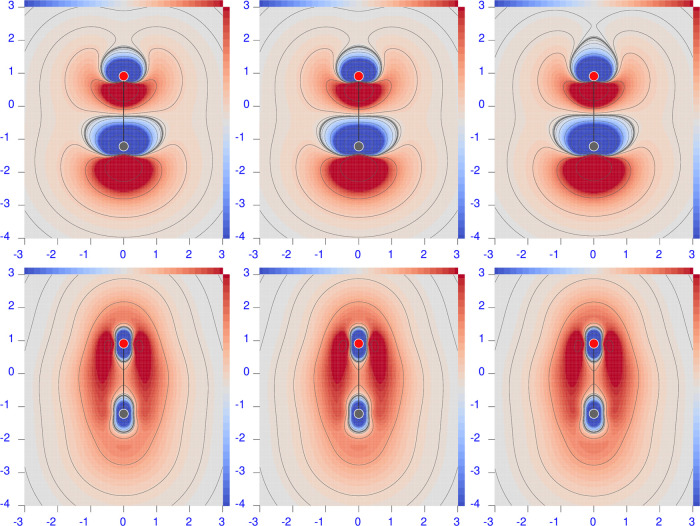
Diverging color map of the origin-independent polarizability density functions of CO. For the three radiation wavelengths λ = 633 (left), 589.3 (middle), and 355 nm (right), the top row shows the perpendicular component, and the bottom row shows the parallel one. The sidebar range is [−0.5(dark blue), 0.5(dark red)] a.u.

Plots of the CCSD origin-independent polarizability density function are shown in Figures 1–6. For all molecules, perpendicular and parallel components of the density  are displayed for three radiation wavelengths in six distinct figure panels; see figure captions for details. The Corey–Pauling–Koltun (CPK) color scheme has been used to distinguish atoms of different chemical elements,^76^ i.e., hydrogen is white (actually light gray), lithium is violet, carbon is dark gray, nitrogen is blue, oxygen is red, and fluorine is green. In all figures, the heaviest and lightest elements are at the top/bottom. Above and to the right of each figure inset, a sidebar is shown that can be used to detect the value of the represented scalar field. The magnitude of any given scalar value, in the light-color intermediate regions, can be obtained as follows: (1) calculate *s* = (*M* – *m*) /32, where *M*/*m* is the max/min value of the sidebar range reported in each figure caption; (2) for each color of interest, count its position (*p*), with respect to *m*/*M*, within the 33 continuous color tiles forming the sidebars; and (3) the scalar value is obtained as *m* + *s*·*p* or *M* – *s*·*p*.^77^

With the exception of LiH, both perpendicular and parallel components do not show significant changes when increasing radiation frequency. The first electronic transition is still too far away for these molecules to appreciate its effect. The LiH molecule exemplifies what happens on approaching the first electronic transition, which is predicted to occur at ∼346 nm, and it is allowed in this case for the parallel component of the electric field. Indeed, a large increment of  at λ = 355 nm can be seen in both positive and negative regions (see the bottom right panel of Figure 2), which is larger for the former, especially in terms of volume extension. The second electronic transition, which allows the perpendicular component, is predicted to occur at ∼270 nm. Its effect can be observed in the plot of  at λ = 355 nm (top right panel of Figure 2). Also, in this case, the red positive region is larger than the negative one, providing after integration the correct general behavior of the electric polarizability tensor components, which increase augmenting the radiation frequency, as documented in Table 3. Comparison with previously reported calculations reveals a fairly good match with the polarizabilities in the dipole length gauge calculated at the same level of theory.^78^ The upward trend of all tensor components upon decreasing the radiation wavelength can be observed. The larger increment is given at a lower λ of 355 nm. At this wavelength, the electric dipole polarizability of LiH is rather large, in particular for the parallel component.

A remarkable feature that can be noted in Figures 2–6 is that  can be negative, compared to its integrated value that is always positive before the first electronic transition. This means that positive density functional domains always prevail over those in which the density is negative. For the molecules considered here, we observe that when present, negative domains are located around a nucleus, whereas positive domains mainly occupy internuclear regions. Second, negative regions are absent around the H atoms and around Li in the ionic salt lithium fluoride (see Figure 4). This is in contrast with the case of LiH (Figure 2), in which the negative domain around the Li nucleus is particularly evident. This counter-polarization can be rationalized by considering the response of the inner electrons (of the ‘heavy’ atoms) as determined by the polarization of the valence electrons rather than directly from the external field. In other words, the induced dipole moment distribution in the valence shell, which is opposite to the external field, induces in turn a counter dipole moment distribution within the inner shell.

**Figure 7:**
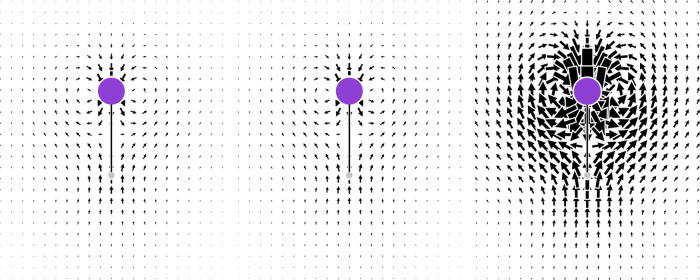
Instant views of the current density induced in LiH by the time derivative of the electric field of the radiation parallel to the symmetry axis of the molecule. Left panel: λ = 633 nm; middle panel: λ = 589.3 nm; right panel: λ = 355 nm.

In terms of eq 23, Figures 1–6 can be interpreted as describing the induced dipole moment density for impinging radiation of a unitary electric field at a time such that cos(*ωt*) = 1. As time passes, one has to consider quite simply that the plots in Figures 1–6 reverse for cos(*ωt*) = −1, with a vanishing polarizability density when cos(*ωt*) = 0. This implies an oscillating electron flux having additional features that can be more properly described by the induced current density vector (eq 24), calculating instant views for different frequencies of the radiation as shown in Figure 7 for the LiH case at cos(*ωt*) = 1. Considering the axial symmetry of the molecule, one can rotate Figure 7 continuously around the molecular axis to obtain a torus-like circulation around the Li atom, flanked along the symmetry axis, above and below the Li atom, by two characteristic conjugated saddle-nodes,^79^ i.e., critical (3, ± 1) stagnation points corresponding to the source and sink of the poloidal flow. This kind of flow has been found at the DFT level to be ubiquitous in atoms and molecules in optical fields.^52^ The inclusion of electron correlation at the CCSD level strengthens this conclusion; see hereafter for a discussion of the effects on the polarizability density due to electron correlation. Increasing the radiation frequency, the toroidal flux gets stronger, keeping its topology, i.e., stagnation point positions, nearly unchanged. This provides a quite stable topological sphere^52^ enclosing the torus. Since the torus is quasi-nonradiative,^80,81^ the electron polarization inside the topological sphere contributes minimally to the Rayleigh scattering of light. Equivalently, it can be said that the contribution to the polarizability of the density inside the topological sphere almost vanishes, keeping the Rayleigh scattering cross section nearly independent of the presence of a toroidal flow.

### 6.3 Electron Correlation Effects on the Polarizability Density

By definition, correlation effects are defined with respect to the HF approximation. In this section, we consider the difference between the CCSD and HF polarizability densities calculated using the same basis set. Owing to the widespread use of DFT, we consider further the difference with respect to B3LYP, which is one of the most popular functionals used so far. In the previous section, we have already noted how the CCSD electric dipole polarizability components, obtained by integrating the corresponding densities, lie somewhere in between the HF and B3LYP results, suggesting under-/overestimation for the former/latter. For the sake of discussion, in the following, we use relative errors (REs) defined as percent absolute deviation from the CCSD results. Our aim is to obtain more detailed information about the correlation effects by observing the differences in the polarizability density distributions.

**Figure 8:**
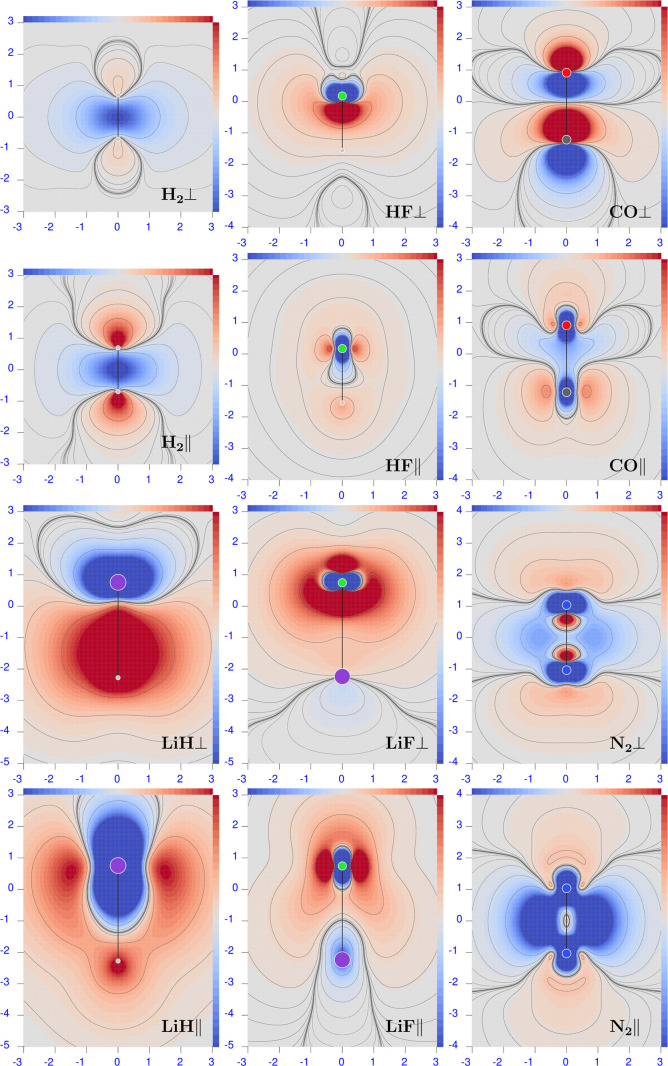
Difference maps of the polarizability density calculated at the CCSD minus HF for λ = 633 nm. Sidebars are ±0.1 a.u., except ±0.01 for H_2_ and ±0.05 for N_2_; red corresponds to positive values and blue to negative values.

**Figure 9:**
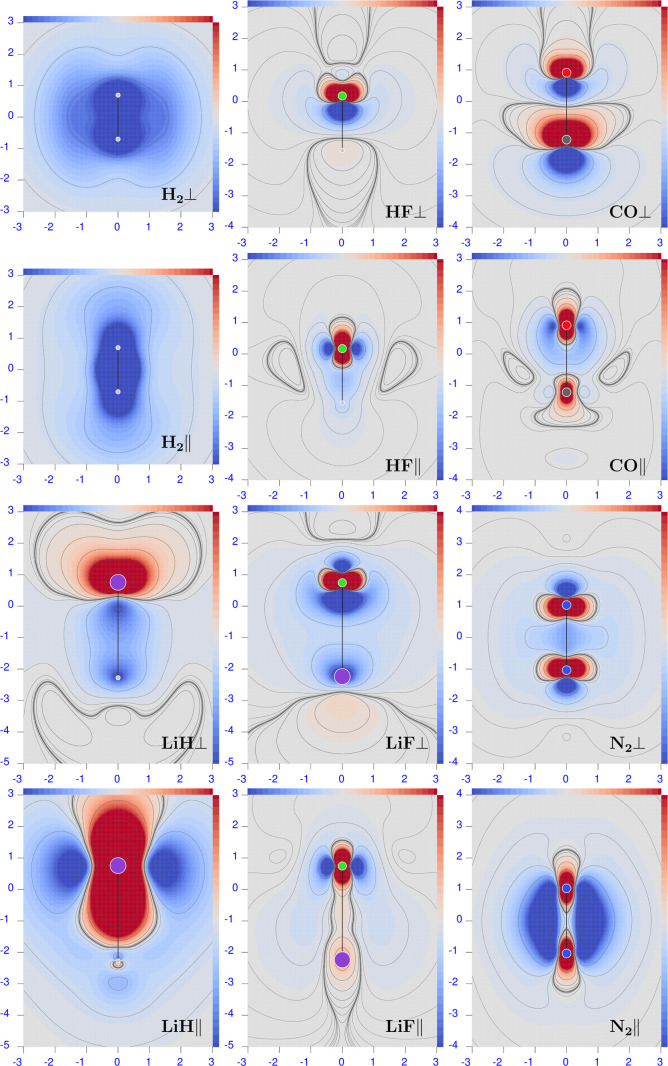
Difference maps of the polarizability density calculated at the CCSD minus B3LYP level of theory for λ = 633 nm. Sidebars are ±0.1 a.u., except ±0.01 for H_2_ and ±0.05 for N_2_; red corresponds to positive values and blue to negative values.

**Figure 10:**
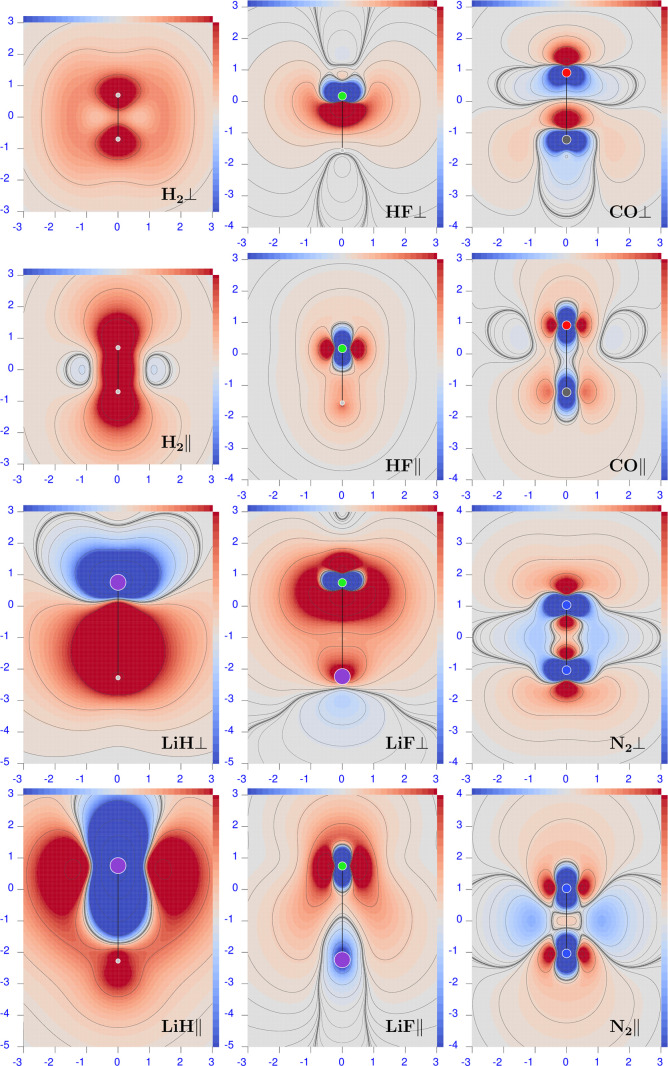
Difference maps of the polarizability density calculated at the B3LYP minus HF level of theory for λ = 633 nm. Sidebars are ±0.1 a.u., except ±0.01 for H_2_ and ±0.05 for N_2_; red corresponds to positive values and blue to negative values.

To this purpose, we report in Figure 8 difference maps of the polarizability density calculated at the CCSD minus HF level of theory, adopting the same basis set and molecular geometries. Figure 9 shows difference maps of the polarizability density calculated at CCSD minus B3LYP, and, for the sake of completeness, Figure 10 displays the difference maps calculated at B3LYP minus HF. To reduce at a minimum the diverging effect of the first electron transition, difference maps have been obtained for the longest radiation wavelength of 633 nm, which is far enough from all predicted first transition energies reported in Tables 1–3.

#### 6.3.1 Hydrogen

For this molecule, we can provide a simple example of how poor the deduction of the effect due to electron correlation can be by comparing polarizability values only. Looking at the polarizability components calculated for λ = 633 nm, reported in Tables 1 and 3, we find an RE of 2.5% for the perpendicular component and 0.9% for the parallel component. Based on these quite small differences, one would suppose that (i) the electron correlation effect is almost negligible on both tensor components and (ii) the perpendicular component is, at any rate, more affected by electron correlation than the parallel one. As shown in the following, both points are not correct.

Inspection of the maps reported in the top and middle left panels of Figure 8, for the perpendicular and parallel components, respectively, reveals the molecular domains where electron correlation plays a major role. The wider of these is the blue one in the bonding region between the two hydrogen nuclei, where both CCSD polarizability density components are lower than HF, accounting for the smaller CCSD integrated quantities. A pair of red domains, where the CCSD polarizability density is larger than HF, can also be seen in the “anti-bonding” regions. These are quite large, especially for the parallel component. Upon integration, there is an evident compensation of the effect, which is more impressive for α_∥_^(*R*,*P*)^. Therefore, contrary to the previously listed points, we conclude that (i) despite the few electrons, the correlation effect is much larger in absolute value than detectable when considering the integrated quantities alone, and (ii) comparatively, the parallel component is more largely influenced by electron correlation.

Both components of the B3LYP polarizability are significantly larger than the CCSD ones; see Table 2. Actually, RE goes up to 11.2 and 6.4% for perpendicular and parallel components, respectively, as documented by the deep blue domains in the maps located in the left top corner of Figure 9.

Consequently, the difference between B3LYP and HF polarizability densities for H_2_ presents only red domains; see the two maps in the left top corner of Figure 10. The B3LYP and HF polarizability densities look similar only for the perpendicular component within the internuclear region; otherwise, they are much different.

#### 6.3.2 Lithium Hydride

In this case, both electric dipole polarizability components are strongly underestimated by the HF method with respect to CCSD; REs are 17.8 and 22.5% for perpendicular and parallel components, respectively.

Looking at the left maps in the last two rows of Figure 8, large red-blue domains of positive/negative differences can be observed. Comparing these maps with those in Figure 2, a similar symmetry can be seen, indicating that the inclusion of electron correlation makes both red and blue domains even more extended with respect to HF. In other words, the toroidal circulation is reinforced by the inclusion of electron correlation. Also, in this case, a large compensation occurs on integrating, which hides electron correlation effects at each point. These are certainly higher than what can be deduced from the integrated polarizability alone. For example, the lower relative error on α_⊥_^(*R*,*P*)^ with respect to α_∥_^(*R*,*P*)^ is due to a greater cancellation, as evident from the difference maps.

The B3LYP polarizability components present REs that are smaller than HF, i.e., 5.6% for the perpendicular component and 18.1% for the parallel component. However, the maps in Figure 9 show large regions of positive and negative differences, revealing that the electron correlation contribution introduced by the CCSD method at each point of the molecular space is by no means comparable with the one given by the B3LYP functional. Cancellation upon integration provides the smaller RE for the perpendicular component, while the excess of the red domain over the blue domain gives the larger RE.

The B3LYP minus HF difference maps for LiH, see the bottom left corner of Figure 10, show, contrary to what one might expect, that the two methods provide polarizability densities that are much more different from each other than they are with respect to CCSD.

#### 6.3.3 Hydrogen Fluoride

Also, in this case, the HF method underestimates both polarizability components with respect to the CCSD. Relative errors are 14.7 and 9.6% for perpendicular and parallel components, respectively. Comparing the difference maps reported in the first two rows of the second column of Figure 8 with those of LiH, we note that they look very different at first glance. However, if we imagine reducing the latter by some factor, then a good resemblance appears, with the red domain for the perpendicular component moved a little toward the fluorine atom. Then, high compensation occurs on integrating also in this case, which masks large electron correlation effects at each point.

The B3LYP functional overestimates both polarizability components with respect to CCSD. Relative errors are reduced to 5.3 and 3.8% for perpendicular and parallel components, respectively. The difference maps reported in the first two rows of the second column of Figure 9 are the negatives of the CCSD minus HF maps. In this case, the dimensions of red and blue domains are nearly equal, canceling each other out more effectively upon integration.

Due to the opposite placement of HF and B3LYP results with respect to CCSD, the difference maps between B3LYP and HF reported in Figure 10 are even more marked.

#### 6.3.4 Lithium Fluoride

For lithium fluoride, the underestimation of the electrical polarizability given by the HF method compared with CCSD is the largest. Relative errors are as large as 24.2 and 23.5% for the perpendicular and parallel components, respectively. Observing the maps in the last two rows of the mid-column of Figure 8, it can be observed that the points where the difference is greatest are found mainly around the fluorine nucleus.

A completely reversed description is given by the difference between CCSD and B3LYP reported in Figure 9, where a large blue domain can be seen. In this case, the REs for the integrated polarizability density are 8.8 and 10.2% for perpendicular and parallel components, respectively. Again, error clearing during the integration process hides the large differences found between the CCSD and B3LYP densities.

#### 6.3.5 Nitrogen

For N_2_, the HF method seems to underestimate a little the perpendicular component of the electric polarizability, while it largely overestimates the parallel component. Relative errors are 0.9 and 9.6% for perpendicular and parallel components, respectively. Looking at the difference maps reported in the last two rows of the right corner of Figure 8, a large negative domain can be seen in the internuclear region, which is very deep and extended, especially for the parallel component, confirming the large RE found. For the perpendicular component, positive domains can be clearly observed, which compensate for the negative contributions. Therefore, the small relative error on the integrated perpendicular component is merely an illusion; point by point, the electron correlation effect is large.

As a rule, B3LYP overestimates both components of the electric polarizability by relative errors of 5.7 and 11.2% for perpendicular and parallel, respectively. Difference maps in Figure 9 show the predominance of blue domains with respect to the red ones.

Even if the B3LYP and HF electric polarizability values seem quite similar for both components, the difference maps in Figure 10 reveal great differences, especially close to N nuclei.

#### 6.3.6 Carbon Monoxide

For CO, the HF method appears to slightly underestimate both polarizability components. Relative errors are 1.7 and 3.8% for perpendicular and parallel components, respectively. Having a look at the difference maps reported in the first two rows of the right column of Figure 8, it can be seen, once again, that the small relative error on the perpendicular component is due to large compensations. In fact, much more extended positive and negative difference domains can be observed near the nuclei of the molecule. In addition, they are larger than those calculated for the parallel component. Point by point, the perpendicular component is more affected by electron correlation effects, which are, however, larger than those observed on the integrated polarizability components.

The B3LYP functional overestimates both polarizability components by relative errors of 6.4 and 3.4% for perpendicular and parallel, respectively. The difference map in Figure 9 looks similar to that in Figure 8 for the perpendicular component and reversed for the parallel component. Again, small relative errors on the integrated values are not sustained by small differences in the polarizability densities, which, in contrast, reveal large discrepancies between CCSD and B3LYP.

## 7 Conclusions

The calculation of the origin-independent dynamic electric dipole polarizability density has been implemented at the CCSD level of theory, thus paving the way for the topological study of the electron polarization induced by optical fields with high accuracy.

Preliminary results for a few simple diatomic molecules have been presented, which show the potential of the method in identifying the regions of the molecular space that make the greatest contributions to the total electric polarizability. Atoms that have electrons in their inner shell show regions of strong counter-polarization near atomic nuclei, which are part of nonradiative toroidal circulations that do not contribute to polarizability. As appears in the cases studied, the polarization density encloses these counter-polarization regions and extends into the internuclear regions, where it is less intense. On increasing the radiation frequency yet remaining below the first electronic transition, the magnitude of the polarizability density increases at each point, and the maps retain similar shapes.

The comparison with the polarizability densities calculated at the HF level allows us to analyze in detail the places of the molecular space where the effects due to electron correlation are more important. In general, the higher the density, the greater the difference between CCSD and HF predictions, i.e., the greater the electron correlation effect. All of the results presented here lead to a single major conclusion: the electron correlation effect on the dipole electric polarizability is much larger than what can be estimated only considering the integrated quantity. The calculated polarizability density using CCSD and HF methods shows, point by point, great differences of either sign, which are compensated upon integration.

Comparison with the polarizability densities calculated at the DFT level allows one to judge how various functionals estimate electron correlation. Here, we have investigated the performance of the B3LYP functional, which systematically overestimates the polarizability density at each point. We are planning to extend this type of comparison to other functionals more suitable for electric response properties while also enlarging the set of reference molecules.

Similar studies are expected to shed light on potentially mismatching ranking of density functionals and eventually contribute to the design of better functionals: functionals, which may be considered poor as they are inadequate to fulfill hypervirial relations,^75^ can nonetheless perform well for integrated properties, since they yield density properties that have large contributions of opposite sign.
